# Characterization of the SGLT2 Interaction Network and Its Regulation by SGLT2 Inhibitors: A Bioinformatic Analysis

**DOI:** 10.3389/fphar.2022.901340

**Published:** 2022-08-15

**Authors:** Zofia Wicik, Anna Nowak, Joanna Jarosz-Popek, Marta Wolska, Ceren Eyileten, Jolanta M. Siller-Matula, Dirk von Lewinski, Harald Sourij, Krzysztof J. Filipiak‬, Marek Postuła

**Affiliations:** ^1^ Center for Preclinical Research and Technology CEPT, Department of Experimental and Clinical Pharmacology, Medical University of Warsaw, Warsaw, Poland; ^2^ Doctoral School, Medical University of Warsaw, Warsaw, Poland; ^3^ Genomics Core Facility, Centre of New Technologies, University of Warsaw, Warsaw, Poland; ^4^ Department of Cardiology, Medical University of Vienna, Vienna, Austria; ^5^ Department of Internal Medicine, Division of Cardiology, Medical University of Graz, Graz, Austria; ^6^ Division of Endocrinology and Diabetology, Interdisciplinary Metabolic Medicine Trials Unit, Medical University of Graz, Graz, Austria; ^7^ Maria Sklodowska-Curie Medical Academy in Warsaw, Warsaw, Poland

**Keywords:** SGLT2, AMPK, mTOR, bioinformatic analysis, network, gene target interaction, prognosis, SGLT2i

## Abstract

**Background:** Sodium–glucose cotransporter 2 (SGLT2), also known as solute carrier family 5 member 2 (SLC5A2), is a promising target for a new class of drugs primarily established as kidney-targeting, effective glucose-lowering agents used in diabetes mellitus (DM) patients. Increasing evidence indicates that besides renal effects, SGLT2 inhibitors (SGLT2i) have also a systemic impact via indirectly targeting the heart and other tissues. Our hypothesis states that the pleiotropic effects of SGLT2i are associated with their binding force, location of targets in the SGLT2 networks, targets involvement in signaling pathways, and their tissue-specific expression.

**Methods:** Thus, to investigate differences in SGLT2i impact on human organisms, we re-created the SGLT2 interaction network incorporating its inhibitors and metformin and analyzed its tissue-specific expression using publicly available datasets. We analyzed it in the context of the so-called key terms ( autophagy, oxidative stress, aging, senescence, inflammation, AMPK pathways, and mTOR pathways) which seem to be crucial to elucidating the SGLT2 role in a variety of clinical manifestations.

**Results:** Analysis of SGLT2 and its network components’ expression confidence identified selected organs in the following order: kidney, liver, adipose tissue, blood, heart, muscle, intestine, brain, and artery according to the TISSUES database. Drug repurposing analysis of known SGLT2i pointed out the influence of SGLT1 regulators on the heart and intestine tissue. Additionally, dapagliflozin seems to also have a stronger impact on brain tissue through the regulation of SGLT3 and SLC5A11. The shortest path analysis identified interaction SIRT1-SGLT2 among the top five interactions across six from seven analyzed networks associated with the key terms. Other top first-level SGLT2 interactors associated with key terms were not only ADIPOQ, INS, GLUT4, ACE, and GLUT1 but also less recognized ILK and ADCY7. Among other interactors which appeared in multiple shortest-path analyses were GPT, COG2, and MGAM. Enrichment analysis of SGLT2 network components showed the highest overrepresentation of hypertensive disease, DM-related diseases for both levels of SGLT2 interactors. Additionally, for the extended SGLT2 network, we observed enrichment in obesity (including SGLT1), cancer-related terms, neuroactive ligand–receptor interaction, and neutrophil-mediated immunity.

**Conclusion:** This study provides comprehensive and ranked information about the SGLT2 interaction network in the context of tissue expression and can help to predict the clinical effects of the SGLT2i.

## Introduction

**FIGURE 1:**
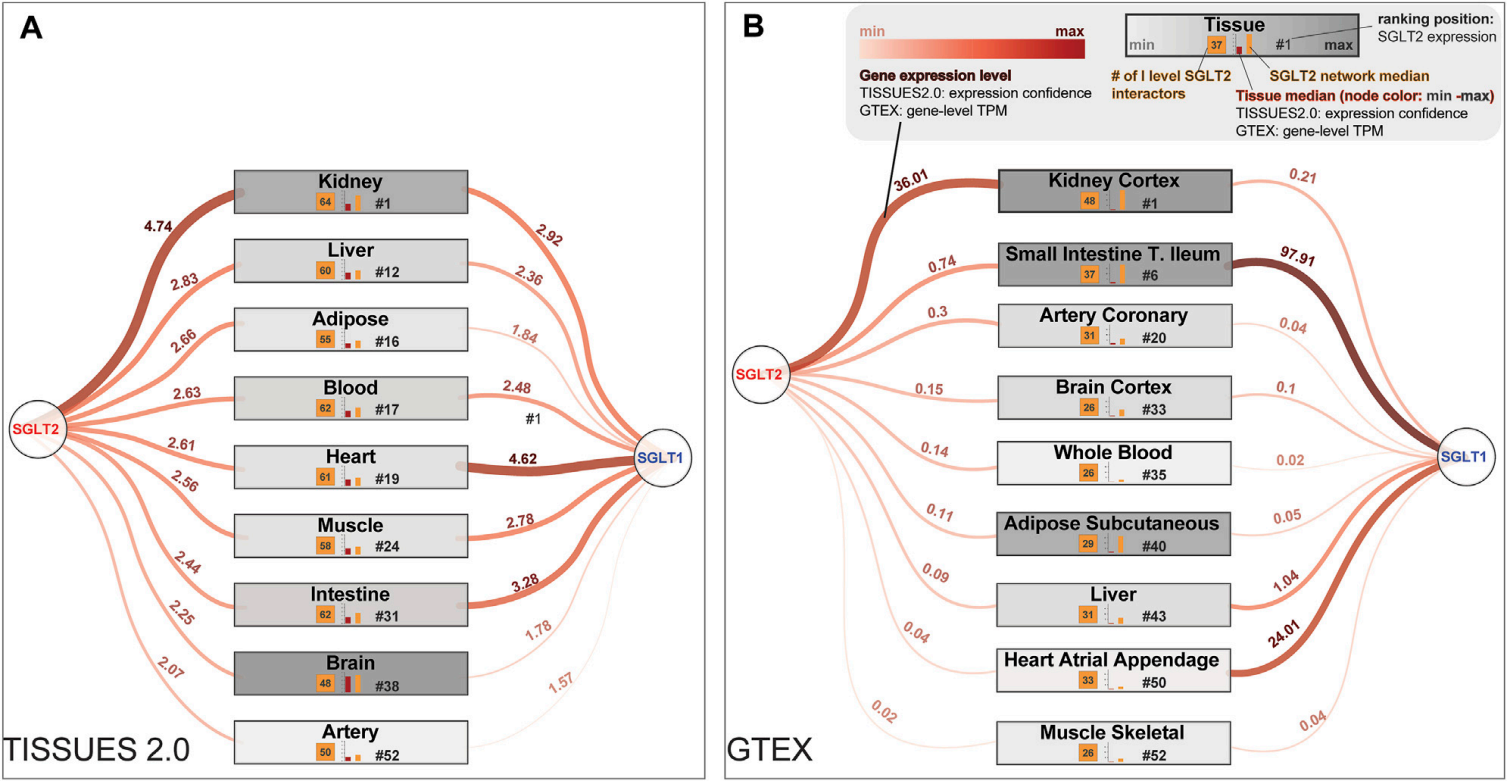
Most interesting tissues from a clinical point of view are sorted by the potential of being affected by SGLT2 and the first-level SGLT2 interaction network. These lists of tissues were generated according to the SGLT2 level, obtained from **(A)** The TISSUES 2.0 database expression confidence values and **(B)** Genotype-Tissue Expression (GTEx) project Transcripts Per Million (TPM) values. In the figure are also present expression levels of SGLT1, which is a co-target for some SGLT2 inhibitors. Legend for both panels is shown in the top right corner of panel **(B)**.

Sodium–glucose cotransporters are six subtypes of the family of receptors, among which two isoforms SGLT1 and SGLT2 are the most abundant. Both are mainly expressed in the renal proximal tubules and less broadly in the urinary tract, excretory glands, and viscus (Figure 1). Sodium–glucose cotransporters are located on the luminal border and enable glucose transport from the luminal filtrate into the proximal tubule’s intracellular space, where they reabsorb some 180 g (1 mol) of glucose from the glomerular filtrate each day (Wright, 2021). The process is led by a sodium gradient, which is caused by a Na⁺/K⁺-ATPase in the membrane, which provides for glucose transport against a concentration gradient. Moreover, several studies reported their expression in most organs and tissues indicating their systemic effect (Wright et al., 2011). Notably, the renal activity of SGLT2 transporters is significantly higher than SGLT1 and it may help to understand their impact on the clinical effects. SGLT2 transporters activation results in glucose reabsorption from the urine back into the bloodstream. Thus, inhibition of SGLT2 transporters increases glucose excretion and leads to lowering its blood levels, which contributed to inventing a novel therapeutic approach. Oral SGLT2 inhibitors (SGLT2i) are rapidly absorbed into the bloodstream, where they remain in the circulation for hours, reducing glucose reabsorption by 50–60% (Wright, 2021). As a result, SGLT2i became a new class of drugs and were primarily established as kidney-targeting, effective glucose-lowering agents used in diabetes mellitus (DM) patients.

Rising evidence indicates that besides renal effects, SGLT2i also have a systemic impact via indirectly targeting heart, endothelial cells, liver, and adipose tissue function by regulating inflammation, oxidative stress, hypoxia, aging, and longevity involved signaling pathways (Hawley et al., 2016; Mancini et al., 2018). Moreover, several studies reported their role in decelerating cancer cells’ expansion via glucose redistribution, diminished cell migration, enhanced apoptosis, and anti-proliferative effect. (Xie et al., 2020; Zhou et al., 2020). SGLT2i lowers plasma glucose levels in a manner independent of insulin secretion, which may result in protective effects on pancreatic beta cells. On the other hand, an increase in glucagon secretion contributes to promoting lipolysis and reducing visceral adipose tissue (Saisho, 2020), and thus treatment with SGLT2i has been shown to reduce body weight (Gager et al., 2021). Moreover, additional benefits of SGLT2i include regression of hepatic steatosis, reduction in serum triglycerides, an increase in high-density lipoprotein (HDL) levels, and uric acid concentration reduction (Packer, 2020a; Szekeres et al., 2021).

For a decade, rising reports emphasized that SGLT2i have a major impact on the cardiovascular (CV) system. The beneficial role of SGLT2 treatment among patients with heart failure (HF) is already confirmed and is demonstrated by a significant decrease in the hospitalizations rate due to HF and CV mortality (McMurray et al., 2019; Gager et al., 2021; Packer et al., 2021). A recent systematic review with a meta-analysis of eight randomized-controlled trials showed that SGLT2i improves cardiovascular outcomes in patients without diabetes mellitus with heart failure. Treatment with SGLT2i in patients without diabetes mellitus improved their metabolic parameters including body weight and blood pressure (Teo et al., 2021). Other meta-analysis showed that treatment with SGLT2i is associated with improved CV outcomes in patients with HF, and the effect of SGLT2 inhibitors on the primary endpoint was independent of underlying diabetes mellitus, age, sex, BMI, renal function, and HF type (Gager et al., 2021). The underlying mechanisms of their action are still unclear, and intensive investigations in this field are conducted.

There are limited studies implementing network analysis to investigate the SGLT2-related interactome. In a recent study, Mai et al. investigated the pharmacological mechanisms of SGLT2i network pharmacology focused on genes associated with DM and HF. They utilized the Swiss Target Prediction tool for the target prediction of selected SGLT2i (canagliflozin, dapagliflozin, empagliflozin, and ertugliflozin) (Mai et al., 2022). They identified 33 core SGLT2i targets including SRC, MAPK1, NARS, MAPK3, and EGFR. Another group investigated the effect of dapagliflozin on diabetic patients with CV disease using a similar Swiss Target (predicted interactions) with molecular docking verification (Yue et al., 2021). In the present study, we decided to use the network medicine approach and *in silico* analysis in order to identify top genes interacting with SGLT2, which were also strongly associated with key terms (autophagy, oxidative stress, aging/senescence, inflammation, AMPK pathways, and mTOR pathways). It seems to be crucial to elucidate SGLT2’s role in a variety of clinical manifestations.

Those key terms were selected based on their important role in CV diseases and the number of publications written by Milton Packer. For example, in the heart, autophagy is important for the turnover of organelles at low basal levels under normal conditions and is upregulated in response to stresses such as ischemia/reperfusion and in cardiovascular diseases such as heart failure (Sciarretta et al., 2018b). Autophagy stimulation and intracellular sodium reduction are considered mediators of the cardioprotective effect of SGLT2i (Packer, 2020a; Packer, 2020b). Oxidative stress defined as excessive production of ROS is strongly associated with HF and malfunction of the NADPH oxidase in diabetes. SGLT2i seem to induce hypoxia- and fasting-like transcriptional processes, which involve the activation of SIRT-1 and HIF-2a signaling (Packer, 2021a). Many systemic metabolic or inflammatory disorders, including diabetes, lead to the development of HF (Paulus and Tschope, 2013; Packer, 2021b). Treatment with SGLT2i shows improvements in metabolic profiles but also inhibition of IL-1β secretion and other pro-inflammatory cytokines, potentially reducing the development of CV diseases in high-risk patients with diabetes (Kim et al., 2020). AMPK pathway plays a key regulatory role in energy homeostasis and is strictly regulated in response to many hormonal and metabolic factors. AMPK phosphorylates proteins involved in the metabolism of fatty acids, cholesterol, carbohydrate, and amino acid and play a role in inflammation, autophagy, mitochondrial function, cell proliferation, and oxidative stress. SGLT2i induce both AMPK and SIRT1, and they have been shown to stimulate autophagy, thereby ameliorating cellular stress and glomerular and tubular injury. Enhanced AMPK/SIRT1 signaling may also contribute to the action of SGLT2i to interfere with sodium transport mechanisms (Packer, 2020c). SGLT2 drugs activate SIRT1/AMPK and suppress Akt/mTOR signaling and, consequently, they can promote autophagy, independent of their effects on glucose or insulin (Packer, 2020d). mTOR pathway in the cardiovascular system regulates both physiological and pathological processes in the heart. It is needed for embryonic cardiovascular development and for maintaining cardiac homeostasis in postnatal life (Sciarretta et al., 2018a). Regarding aging and senescence, SGLT2i appear to be promising in the treatment of aging-related diseases, due to their regulation of multiple longevity pathways, including above mentioned, closely resembling calorie restriction, and their established efficacy in reducing cardiovascular events and all-cause mortality (Hoong and Chua, 2021). We further selected the associated gene list using publicly available ontological databases. In this study, we also characterized the tissue-specific influence associated with targeting the SGLT2 interactors by common SGLT2i.

## Materials and Methods

### Selection of Key Term-Related Gene Lists

To identify genes associated with key processes (autophagy, oxidative stress, aging/senescence, inflammation, AMPK pathways, mTOR pathways) we performed a screening of the Reactome and the KEGG pathway databases looking for the related ontological terms. We also selected aging-related genes from GenAge and Longevity Map databases (https://genomics.senescence.info/download.html) (Budovsky et al., 2013; Tacutu et al., 2017). Additionally, we identified genes associated with selected biological processes using the Gene Ontology database through the biomaRt R package (Durinck et al., 2009) for the following keywords: autophagy, oxidative stress, and senescence. A complete list of ontological terms and associated genes with sources is available in Supplementary File S2.

### Construction of the Interaction Network Model

SGLT2 interaction network was constructed and visualized by retrieving the first level SGLT2 interactors (direct interactors) from the complete Human interactome version 11.0b (17 October 2020 to 12 August 2021), stringApp v1.5.1 (Doncheva et al., 2019) through Cytoscape software v3.8.2 (Shannon et al., 2003), which is a free, open-source, visual interface for importing, visually exploring, and analyzing graphical data including molecular networks. It allows efficient expression of the interactions between protein and protein, protein and DNA or gene, in order to visualize network relationships. Next, the network was expanded by retrieving second-level SGLT2 interactors (indirect interactors) from the human interactome. It was done by mapping all of the neighbor nodes of the first-level SGLT2 interactors. A complete list of genes involved in the SGLT2 network is available in Supplementary File S2.

### Drug Repurposing

Drug repurposing analysis was performed in R using the Binding Database (Dataset BindingDB_All_2021m9.tsv.zip was downloaded from https://www.bindingdb.org/bind/chemsearch/marvin/SDFdownload.jsp?all_download=yes using update version from 2021-10-01) (Liu et al., 2007). For further analyses, we selected ligands targeting human genes, which had at least one value associated with enzyme inhibition constant Ki (nM) or the half-maximal inhibitory concentration IC50 (nM). Gene symbols were unified using the NCBI human annotation file. In further steps, we performed prioritization of the ligands based on their ability to target SGLT2, and its first- and second-level interactors. For gene annotation and ligand prioritization, we used wizbionet R package (Wicik et al., 2021).

### SGLT2-Related Tissue-Specific Expression

Tissue-specific expression of SGLT2 was retrieved from the TISSUES 2.0 database using download “all channels Integrated” for human organism (https://tissues.jensenlab.org/Downloads) (Palasca et al., 2018) and from the GTEX database (median gene-level TPM by tissue). Human-related tissues were filtered out from the TISSUES 2.0 using BRENDA tissue ontology related to the whole organism term (Gremse et al., 2011). Ranking of the tissues in terms of overall high levels of SGLT2, SGLT1, and first-level SGLT interactors median expression, and a number of first-level SGLT2 interactors was performed by division of each numerical column into four clusters using OneR algorithm (https://cran.r-project.org/web/packages/OneR/index.html) implemented in wizbionet R Package (Wicik et al., 2021).

### Shortest Pathway Analysis

Shortest pathway selections were performed using PathLinker Cytoscape App (Gil et al., 2017) using the following settings: unweighted and undirected network, Target- SGLT2, Sources-genes associated with a given key term (autophagy, oxidative stress, longevity, aging/senescence, inflammation, AMPK pathways, and mTOR pathways). We analyzed the top 50 paths and further for visualization selected the top 10 paths associated with each key term.

### Enrichment Analysis

Enrichment analysis of diseases (DisGeNET), pathways (Bioplanet_2019), biological processes (GO_Biological_Process_2021), and the KEGG pathways (KEGG_2019_Human) was performed using the EnrichR REST API from the R level (Chen et al., 2013). EnrichR provides analytical access to more than 190 ontological databases https://maayanlab.cloud/Enrichr/#libraries. In all analyses, BH adjusted *p*-value cutoff was set as lower than 0.05. Visualization of ontological results was performed in R using ggplot2 and ggrepel libraries.

## Results

### SGLT2 Interaction Network Model Construction

**FIGURE 2:**
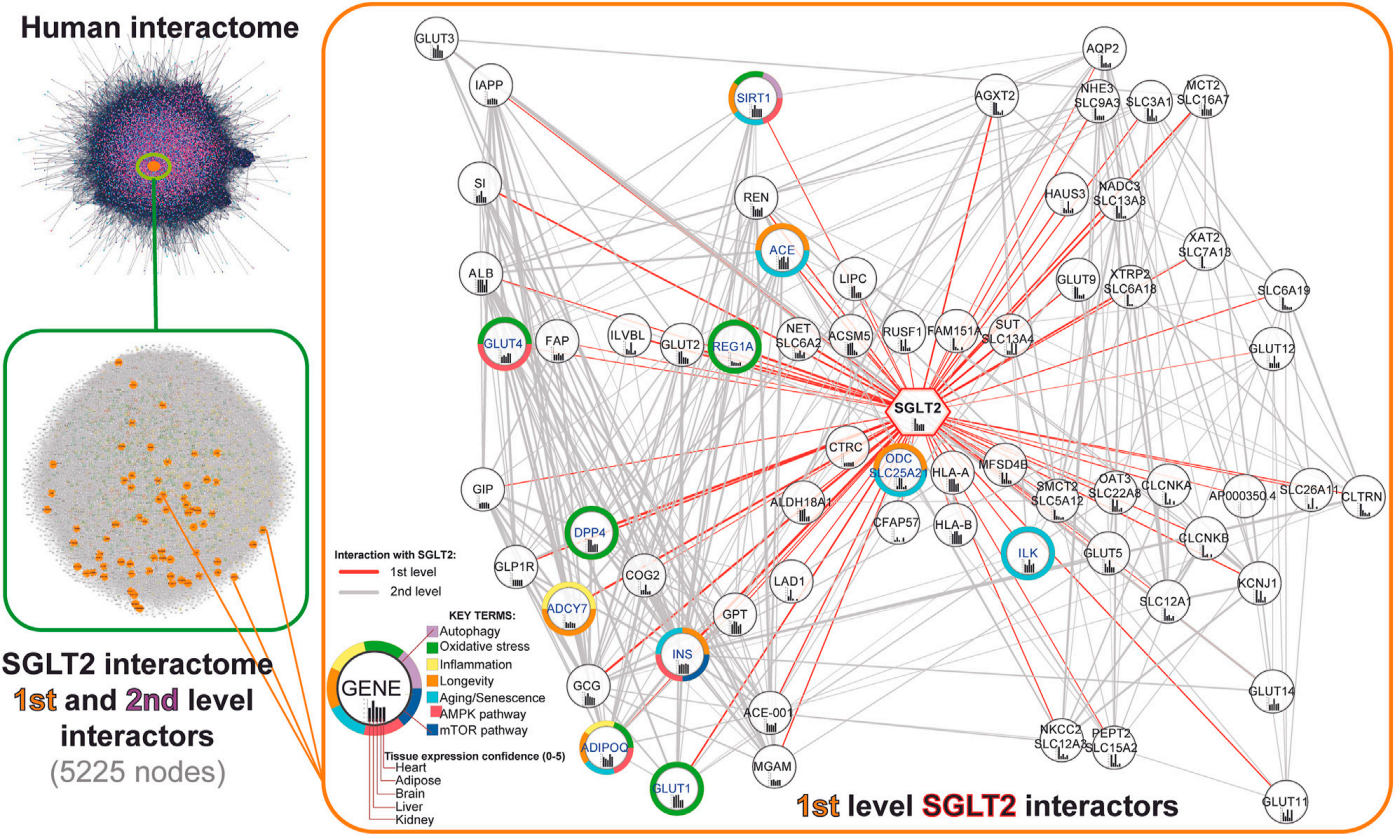
Workflow of the SGLT2 interaction network retrieval from the human interactome. The first level SGLT2 network is visualized using a hierarchical layout, predicting the signal flow from the top to the bottom. Genes associated with specific key terms have blue labels.

In order to recreate the SGLT2 interaction network, we first retrieved all first-level SGLT2 interactors from the human interactome (stringApp v1.5.1). Furthermore, we expanded it by including second-level interactors by mapping all the neighbor nodes of the first-level SGLT2 interactors. In total, we obtained a network of 5225 nodes, including SGLT2 and its 65 first-level interactors and 5160 second-level interactors. Next, we performed visual mapping of gene annotations related to key terms (autophagy; oxidative stress; inflammation; longevity; aging; AMPK pathway; mTOR pathway). The schematic workflow of constructing the SGLT2 interaction network and first-level SGLT2 interactors are shown in Figure 2. All genes included in the network are available in Supplementary File S2.

### Tissue Expression Analysis of SGLT2 and SGLT1 in SGLT2-Related Network

To identify tissues most affected by the alterations in the SGLT2 interaction network, we mined the TISSUES and GTEX databases. We selected tissue expression confidence scores for SGLT2 and calculated mean expression confidence scores for the first-level SGLT2 interactors. We included only those interactors whose expression was ≥25% of median tissue expression. We also paid special attention to the following tissues affected by SGLT2-related treatment: kidney, liver, adipose tissue, blood, heart, muscle, intestine, brain, and arteries. The results of this analysis using the TISSUES database are shown in Figure 1. All top-ranked tissues identified in this analysis are shown in Supplementary File S1.

In terms of overall high levels of SGLT2, SGLT1, and first-level SGLT interactors median expression, and a number of first-level SGLT2 interactors we observed the following order in the TISSUES 2.0 database: kidney, liver, blood, intestine, heart, muscle, adipose tissue, brain, and artery. While in the GTEX database it was: kidney cortex, small intestine terminal ileum, adipose subcutaneous, heart atrial appendage, heart left ventricle, brain cerebellum, artery coronary, liver, brain cortex, whole blood, and muscle-skeletal.

The highest expression confidence of SGLT2 among our tissues of interest was observed for the kidney, liver, and adipose tissue according to the TISSUES database. It reflected the results from the GTEX database, where the kidney cortex showed the highest transcripts per million (TPM) values. Additionally, for the kidney, we observed expressions of 64 from 66 first-level SGLT2 interactors identified by network analysis.

In terms of SGLT2 expression, heart tissue was in 19th place, and artery in 52nd place according to the TISSUES database; accordingly artery coronary and heart atrial appendage were located in 20th and 50th place, respectively, in the GTEX dataset. Liver tissue had high expression confidence (14th place) only in the TISSUES database, while in GTEX it was in 43rd place. According to the TISSUES database, brain tissue was located in the 40th place. In the GTEX dataset, we observed high expression levels of SGLT2 in the brain cerebellum (fifth place, Supplementary File S1), while the brain cortex was located in 33rd place. Adipose tissue was located in the 17th place with 51 genes according to the TISSUES database, while according to GTEX it was on 40th and 41st.

Analysis of expression levels of the SGLT1 using the TISSUES 2.0 database was highest in the heart, intestine, and kidney. While in the GTEX database it was small intestine terminal ileum, heart atrial appendage, and liver. In the extended analysis using the GTEx database we also observed strong expression of SGLT1 in the minor salivary gland and skin. In terms of high expression of both SGLT2 and SGLT1 kidney and heart, tissue was highest in the ranking.

### Drug Repurposing and Prioritization Analysis Within SGLT2 Network

**FIGURE 3:**
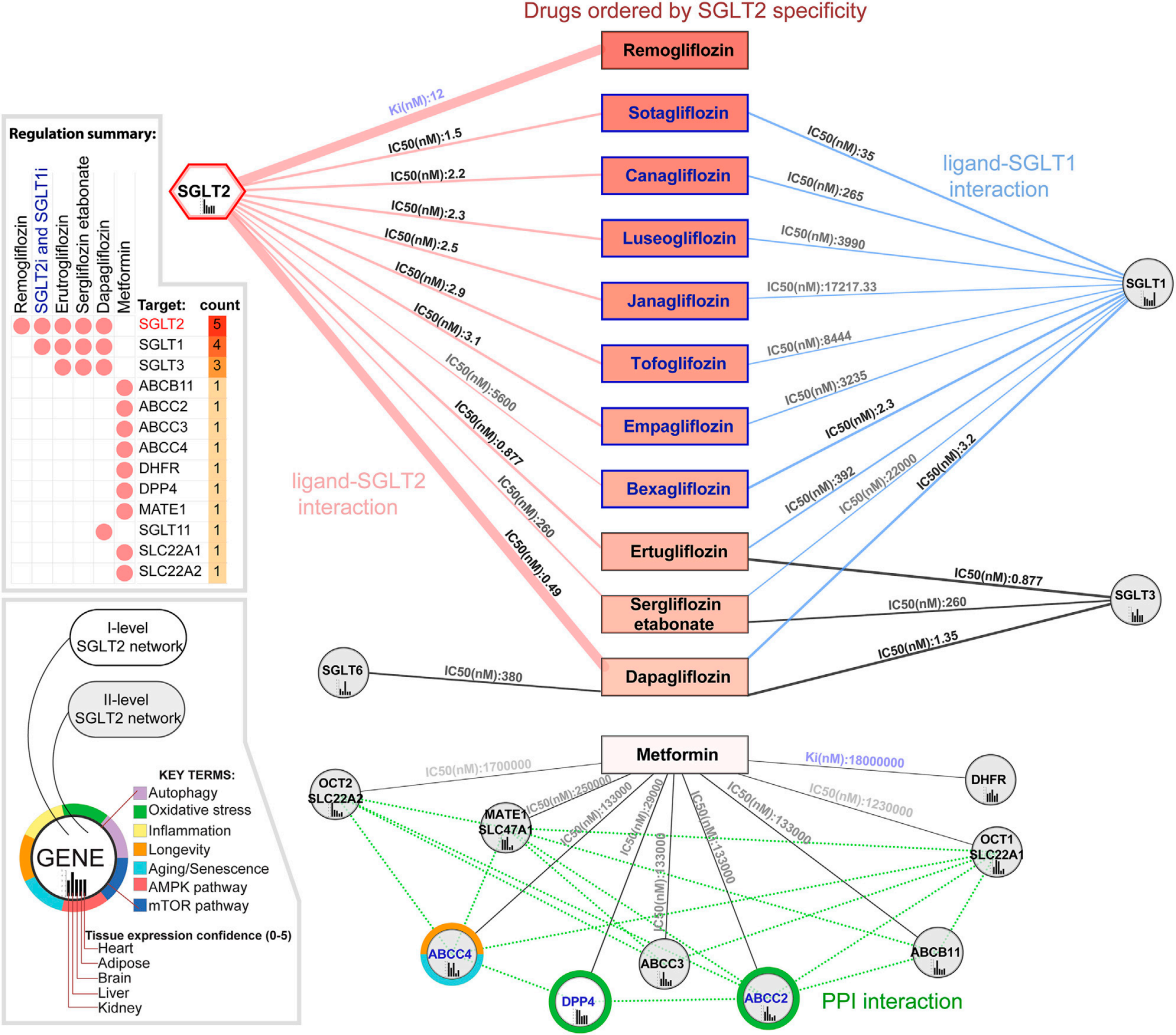
Drugs modulating the SGLT2 and its network. In the figure are presented known SGLT2 ligands ordered vertically by increasing enzyme inhibition constant Ki (nM) and half maximal inhibitory concentration IC50 (nM). Additionally, we included metformin, not targeting SGLT2 but regulating other components of its network. Ki reflects the binding affinity, the smaller the Ki, the greater the binding affinity, and the smaller amount of medication needed in order to inhibit the activity of that enzyme. IC50 reflects the functional strength of the inhibitor for a drug, is dependent on the enzyme concentration, and is always larger than Ki. Genes associated with specific key terms have blue labels.

Analysis of the SGLT2-drug interactions in the context of the SGLT2 network identified 1571 chemical compounds interacting with SGLT2 according to the Binding Database. In the drug repurposing and prioritization analysis we took into account two parameters; enzyme inhibition constant Ki (nM), and the half-maximal inhibitory concentration IC50 (nM). We identified 1570 ligands interacting with SGLT2, 771 of them were interacting with at least one other gene from the SGLT2 network. The analysis focused on known ligands' specificity toward SGLT2 and identified remogliflozin as the most specific according to its Ki value, regulating only SGLT2. Known ligands (which had common names in the Binding DB) with the lowest IC50 values towards SGLT2 were sotagliflozin, canagliflozin, and luteogliflozin. Those drugs also targeted SGLT1. From all the drugs binding with both, SGLT2 and SGLT1, Bexagliflozin showed the lowest IC50 towards this SGLT1. Empagliflozin was located in seventh place, strongly targeting SGLT2 (IC50 = 3.1) and weakly SGLT1 (IC50 = 3235), while dapagliflozin was placed in 11th place in terms of SGLT2 specificity. Dapagliflozin, in comparison with other drugs, showed the strongest regulation of SGLT2 (IC50 = 0.49), while its regulation of SGLT1 (IC50 = 3.2) was in second place after bexagliflozin; SGLT3 was in second place after ertugliflozin (IC.50 = 1.35). Dapagliflozin was also the only drug regulating SGLT6 (IC50 = 380). Analysis of metformin targets within the SGLT2 network identified nine genes but interactions with them had a low affinity. Only DPP4 was present among first-level SGLT2 interactors. The results of this analysis are shown in Figure 3 and Supplementary File S1 (Supplementary Figure S2, Supplementary Table S1).

### Analysis of Drug Interactions in the Context of Tissue Expression of Their Targets

Analysis of drug interactions in the context of tissue expression of their targets pointed out the influence of SGLT1 regulators (positions 2–11) on heart tissue (tissue expression confidence = 4.617/5). While regulators of SGLT3 such as ertugliflozin, dapagliflozin, or sergliflozin etabonate seem to also have a stronger impact on brain tissue (tissue expression confidence = 4.34). For dapagliflozin, it also includes potential regulation of also expressed in brain SGLT6 (4.519). Analysis of tissues affected by analyzed drugs with more targets than SGLT2 and SGLT1 revealed that the top tissues targeted by, in this case ertugliflozin and sergliflozin etabonate, were as follows: intestines, kidney, heart according to both databases, and additionally adipose tissue according to the GTEX database. While the top targets for dapagliflozin was the kidney, intestine, and heart. For metformin, according to both databases, the top targets were liver in kidney tissue. Additionally in the GTEX database, we observed the strong influence of dapagliflozin on the brain cortex. A complete tissue ranking for selected drugs in the context of our tissues of interest and all analyzed tissues is shown in Supplementary File S1 as Supplementary Figure S2.

### Shortest Path Analysis to Identify SGLT2 Interactors Regulating Analyzed Key Terms

**FIGURE 4:**
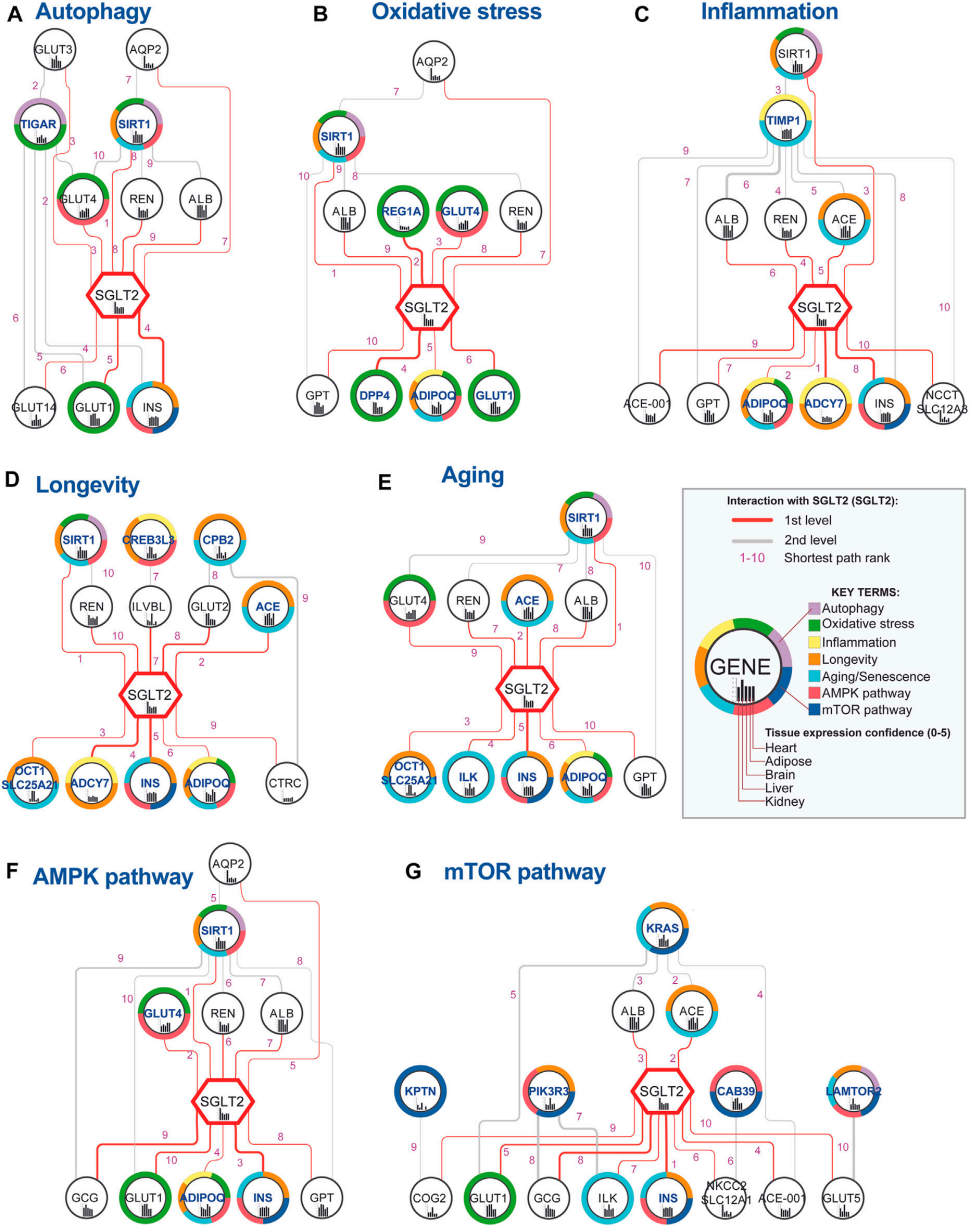
Top 10 shortest paths from 50 identified between SGLT2 (SLC2A5) and each key terms-related gene. Genes associated with specific key terms **(A–G)** have blue labels. The shortest path analysis was performed using the PathLinker Cytoscape app (Paths ranks are marked with pink numbers). Notice that interactions between SGLT2 and SIRT1 had very low ranks for all analyzed key terms, except for mTOR. The thickness of the edges is related to the interaction confidence level obtained from the String database.

In order to identify nodes that could play a crucial role in SGLT2 signaling, we analyzed paths between SGLT2 neighbors associated with analyzed key terms and the SGLT2 transporter. The networks generated in this analysis are shown in Figure 4. Shortest paths analysis identified SIRT1-SGLT2 among the top five interactions across six from seven analyzed networks associated with the key terms. In all networks, except for mTOR, it was present in the top five shortest paths.

Interactions with SGLT2, which had the highest occurrence in networks and best path rank were sirtuin 1 (SIRT1)-SGLT2, SGLT2-insulin (INS), renin (REN)-SGLT2, albumin (ALB)-SGLT2, and SGLT2-adiponectin (ADIPOQ). Interactions between SGLT2-INS, REN-SGLT2, and ALB-SGLT2 were present among the top 10 paths in six networks. Additionally, SGLT2-ADIPOQ was present among the top 10 paths in five networks, and ACE-SGLT2 was present among the top five paths in four networks. Within the top 10 shortest pathways associated with key terms following genes targeted by known drugs: SGLT2, DPP4, and GLUT1. GLUT1 according to the String database is a potential interactor of SGLT1 (edge score 0.724/1).

### Identification of the Top SGLT2 Interactors

In order to identify the top SGLT2 interactors, we combined, aggregated and ranked obtained results from shortest paths analysis (Table 1). This analysis pointed out SIRT1 as the most important SGLT2 interactor in all analyzed networks associated with six key terms. Other genes related to key terms and present in all seven top 10 shortest path analyses related to them were adiponectin (ADIPOQ), INS (insulin), GLUT4, ACE, and GLUT1. We also identified multiple genes present in all seven top 10 shortest path analyses but not present on gene lists related to analyzed key terms, but potentially associated with them: GPT, GCG, and ALB. Other highly represented genes in shortest paths analysis were ILK, ADCY7, COG2 (first-level interactors with SGLT2), and MGAM (second-level interactor with SGLT2). The ranked list of top SGLT2 interactors is shown in Table 1.

**TABLE 1:** Top genes in the context of the level of their interaction with SGLT2, association with key terms (proc.), and presence in shortest paths analysis (net) for top 10 and 50 pathways. If a gene was associated with a key term and was present in shortest paths analysis was marked as “both”. This approach enabled us to identify interesting genes, which could play a role in SGLT2-related regulation and be involved in key terms/processes. Genes were ordered based on their association with the key terms and presence in the top 10 shortest path analyses. On the table are shown genes that appeared in at least two top 50 shortest path analyses. Tissue expression confidence levels (0-5) were retrieved from the TISSUES 2.0 database.

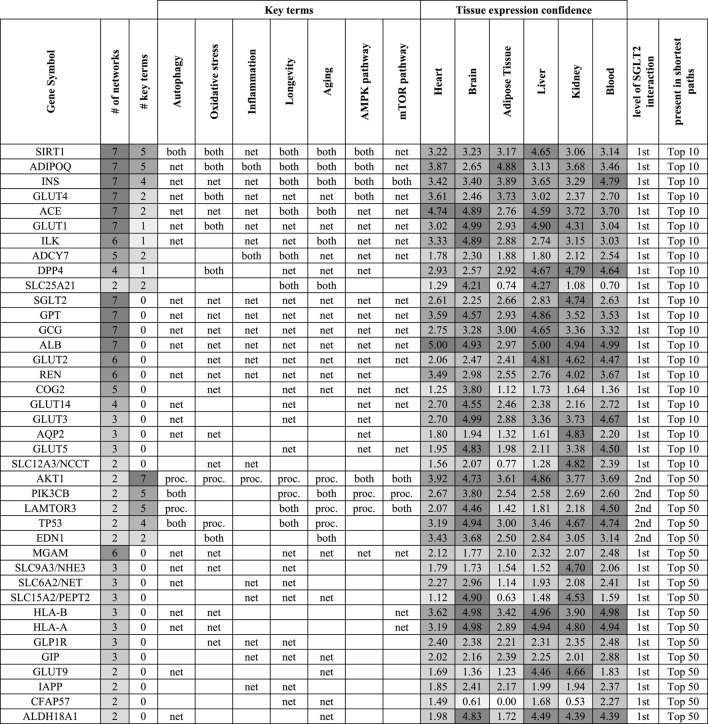

### Pathway Enrichment Analysis of SGLT2 Network Components

**FIGURE 5:**
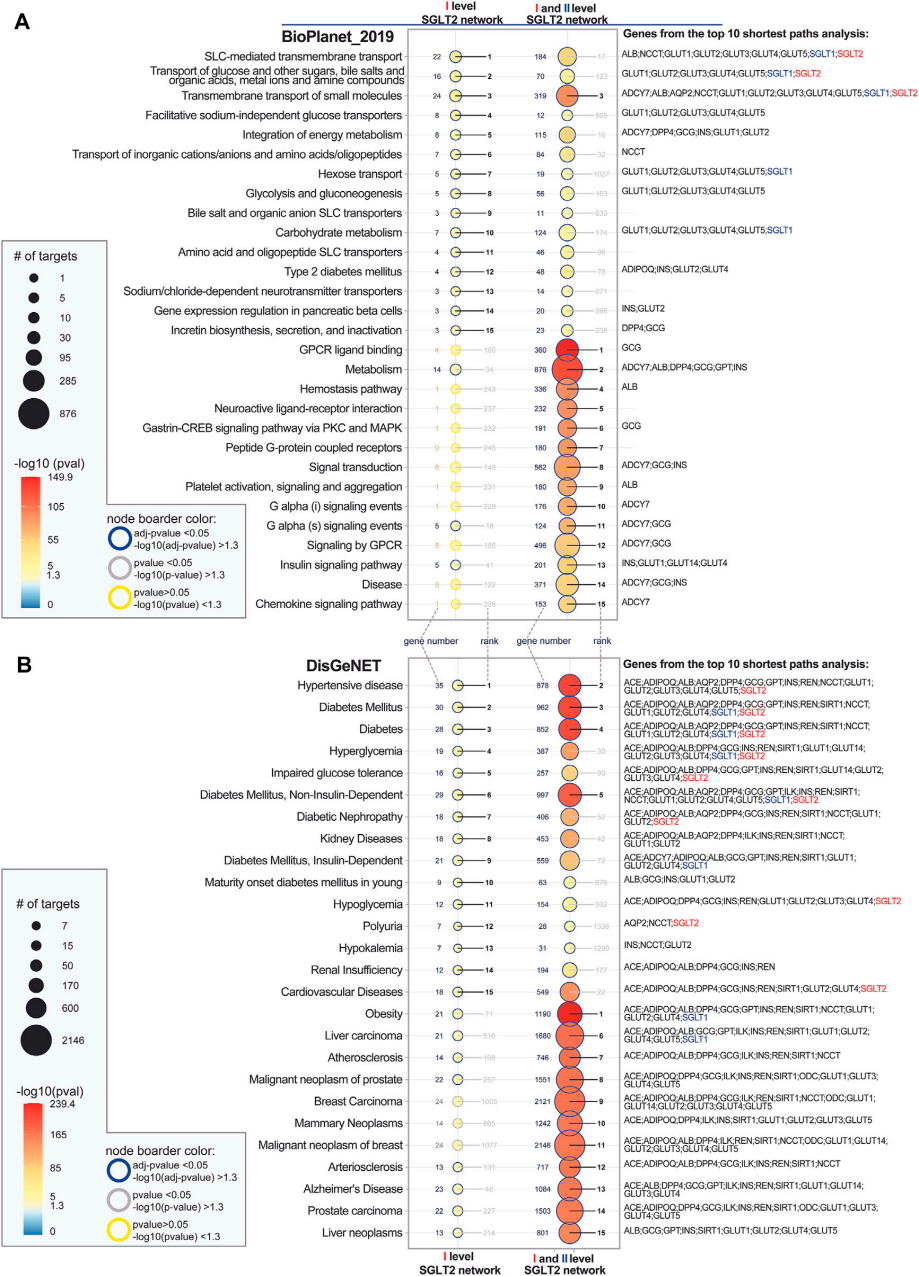
Top 15 significantly enriched pathways **(A)** and diseases **(B)** associated with first level SGLT2 network and first and second level SGLT2 interactors. Analysis was performed using EnrichR API using the following databases Bioplanet_2019 (pathways), DisGenet (diseases). The enriched terms were ordered by the level of significance for the first level SGLT2 network and then for the extended interaction network. SGLT2 (SLC5A2) is marked with red color, and SGLT1 (SLC5A1, second-level interactor) with blue color. The adjusted *p*-values show categories, which are more likely to have biological meanings. The color gradient of the dots is associated with corresponding adjusted *p*-values. Red color indicates low *p*-values (high enrichment), and blue indicates high *p*-values (low enrichment). The size of the dots is associated with the number of enriched genes.

In order to identify potential modifiers of the SGLT2 network, we performed a series of enrichment analyses (Figure 5). Pathway enrichment analysis showed that both first-level network components have the strongest association with transport-related pathways and energy metabolism. Analysis of the extended SGLT2 network (combined first and second-level SGLT2 network) additionally showed the high significance of pathways related to G-protein-coupled receptors (GPCRs) ligand binding, metabolism, hemostasis pathway, and platelet signaling, activation, and aggregation. On the other hand, additional analysis using the KEGG pathway database did not show a strong overlap between first-level network components and extended networks except for cAMP signaling (Supplementary Figure S3B in Supplementary File S1). In this second analysis, the insulin-related pathways were more enriched in the first-level network, while in the extended network we observed strong enrichment of neuroactive ligand–receptor interaction and pathways involved in cancer and pathways associated with atherosclerosis.

### Disease Enrichment Analysis of SGLT2 Network Components

Disease enrichment analysis showed the highest overrepresentation of hypertensive disease, DM-related diseases for both levels of interactors. Additionally, for the extended SGLT2 network, we observed enrichment in obesity, atherosclerosis and arteriosclerosis, breast cancer-related diseases, and Alzheimer’s disease. Additional analysis of the biological processes associated with SGLT2 networks showed as expected strong enrichment of glucose and hexose transmembrane transport for the first-level interactors. Analysis of the extended network showed on the other hand overrepresentation of processes related to the regulation of inflammatory response and neutrophil-mediated immunity (Supplementary Figure S3B in Supplementary File S1).

### Overlap of Enrichment Analysis With SGLT2 Ligand Interactions

Overlap of enrichment analysis with SGLT2 ligands interactions pointed out three pathways: Transmembrane transport of small molecules, SLC-mediated transmembrane transport, transport of glucose and other carbohydrates) and four disease terms (diabetes mellitus, diabetes, hyperglycemia, diabetes mellitus non-insulin-dependent), which are associated with ligands of both, SGLT2 and SGLT1. (Supplementary Table S1 in Supplementary File S1).

Analysis of ligands targeting other components of the SGLT2 network besides SGLT2 or SGLT1 revealed that ertugliflozin, sergliflozin etabonate, and dapagliflozin have additional targets associated with hypertensive disease, diabetes mellitus, diabetes, hyperglycemia, impaired glucose tolerance, diabetes mellitus, non-insulin-dependent, diabetic nephropathy, and hypoglycemia. The analysis focused on dapagliflozin identified all four targets as associated with diabetes mellitus (SGLT1; SGLT6; SGLT2; SGLT3); while ertugliflozin and sergliflozin were regulating three of them (SGLT1; SGLT2; SGLT3).

For metformin, the highest number of its targets were associated with malignant neoplasm of the prostate (six genes) and breast carcinoma (seven genes: ABCB11; ABCC2; ABCC3; ABCC4; DHFR; DPP4; SLC22A1).

## Discussion

In this study, we investigated the effect of alteration of the SGLT2 interaction network caused by SGLT2i in order to explore its consequence for tissues and potential influence on disease-related phenotypes. Reconstruction of the SGLT2 network enabled us also to evaluate SGLT2i based on their specificity toward SGLT2 and point out potential side effects of their usage on different tissues, phenotypes, and pathways.

### Interpretation of Tissue Expression Analysis of SGLT2 and SGLT1

Analysis of tissue-specific expression of SGLT2 showed the highest expression confidence of SGLT2 in the kidney tissue. Additionally, we observed expressions of 64 from 66 first-level SGLT2 interactors identified by network analysis. This result reflects the literature data, which shows the highest SGLT2 expression in the kidney cortex, where the expression was approximately 300-fold higher than the tissue with the next highest level of expression, the kidney medulla (Chen et al., 2010). Literature data also shows that SGLT2 mRNA and protein expression is increased in renal biopsies from human subjects with diabetic nephropathy (Wang et al., 2017).

Our prioritization analysis identified the intestine in a high position in terms of SGLT2 expression according to both analyzed expression databases. We also observed for intestines, but also adipose tissue the highest difference in terms of SGLT2 first-level interactors expression versus tissue median expression, which points them out as an especially important target for SGLT2i. In intestines, but also heart tissue we also observed high expression confidence of SGLT1. In the intestines, SGLT1 plays a role in glucose uptake and it might play an important role in T1DM and type 2 diabetes mellitus (T2DM) therapy (Rieg and Vallon, 2018).

In the extended analysis using the GTEX database, we also observed strong expression of SGLT1 in the minor salivary gland and skin. Serious skin and subcutaneous tissue disorders were reported in the context of ipragliflozin and dapagliflozin treatment (Reich et al., 2009; Sakaeda et al., 2018). The dysfunction of salivary glands occurs as a complication of diabetes (Aitken-Saavedra et al., 2015). In diabetic rats simultaneously with reduced sympathetic outflow to salivary glands, the reduced translocation of SGLT1 to the cell membrane was observed, which indicates the role of the protein in the function of this gland (Sabino-Silva et al., 2010).

While there are publications evaluating SGLT genes expression levels in different tissues, to our knowledge, there is no analysis investigating the expression levels of such a high number (66) of first-level SGLT2 interactors. Taking into account that many of the SGLT2i target SGLT2, but also SGLT1 (dapagliflozin, empagliflozin) and other genes, this kind of analysis can help predict the additional reactions of those drugs in other tissues.

### Interpretation of the Drug Repurposing and Prioritization Analysis Within the SGLT2 Network

Our study identified remogliflozin as the strongest acting SGLT2i according to its Ki value, and dapagliflozin in terms of its IC50 value (Figure 3). Additionally, dapagliflozin also targets SGLT1 (IC50 = 3.2, highest expression in intestine and heart), SGLT3 (IC.50 = 1.35, highest expression in brain), and SGLT6 (IC50 = 380, highest expression in brain). Other inhibitors such as ertugliflozin and sergliflozin etabonate seem to also have a potential impact on brain tissue, but only through SGLT3. SGLT3-mediated glucose entry stimulates an electrical activity on the neuronal membrane. This can either cause hyperpolarization or excitatory depolarization in the hypothalamus, in which neurons have glucose-sensing ability (Burdakov et al., 2005).

Additionally, we analyzed the effect of metformin on the SGLT2 network, as clinical guidelines suggest the use of SGLT2i (canagliflozin, dapagliflozin, and empagliflozin) as add-on therapy in patients for whom metformin alone does not achieve optimal glycemic control, or as initial dual therapy with metformin in patients who present with higher glycated hemoglobin (HbA1c) levels (Sonne and Hemmingsen, 2017; Donnan and Segar, 2019). Both metformin and SGLT2i can cause rare but serious life-threatening complications including lactic acidosis (with metformin) and euglycemic diabetic ketoacidosis (with SGLT2i) (Donnan and Segar, 2019). Our analysis did not show overlap in metformin targets with analyzed SGLT2i but identified nine other metformin targets present among first and second-level SGLT2 interactors. One of them, dipeptidyl peptidase 4 (DPP-4) highly involved in glucose and insulin metabolism, as well as in immune regulation through lymphocytes T activation was identified by us as the top SGLT2 interactor in the shortest paths analysis associated with sub-networks related to oxidative stress, longevity, aging, and AMPK pathway. Interestingly the information about metformin targeting DPP-4 is scarce, and mostly points out co-treatment of a combination of DPP-4 inhibitors (gliptins) and metformin in diabetes and/or mild to moderate hypertension (Mistry et al., 2008; Scheen, 2017).

### Interpretation of the Analysis of Drug Interactions in the Context of Tissue Expression of Their Targets

Our analysis of tissue expression of drug targets showed the influence of common SGLT2i targeting SGLT1 regulators on heart tissue (tissue expression confidence = 4.617/5), after intestine and kidney. Interestingly, dapagliflozin was described to have a stronger impact on kidney tissue than other analyzed SGLT2i (Heerspink et al., 2020; Gager et al., 2021). In addition, we observed SGLT2i influence on adipose tissue. It was recently highlighted that adipose tissue is a target for canagliflozin, which ameliorated diet-induced obesity by increasing intra-adipose sympathetic innervation (Yang et al., 2021). Also, empagliflozin reduces epicardial adipose tissue in nondiabetic patients with HFrEF (Requena-Ibáñez et al., 2021). Our results may support above mentioned observations showing that SGLT2i has the ability to directly regulate adipocyte activity, not only indirectly through regulation of insulin metabolism or inflammation pathways.

### Interpretation of the Shortest Path Analysis to Identify SGLT2 (SLC5A2) Interactors Regulating Analyzed Key Terms

#### Sirtuin 1 and Sirt6 as Regulators of the SGLT2 Interaction Network

We identified SIRT1-SGLT2 interaction among the top five interactions across six from seven analyzed networks associated with the key terms (Figure 4; Table 1). In all networks, except for mTOR, it was present in the top five shortest paths. SIRT1 was also identified as the top SGLT2 interactor, appearing in all analyzed networks related to key terms. The shortest path analysis and hierarchical layout on the complete SGLT2 network also pointed out that the most likely normal signal flow is from SIRT1 to SGLT2. This finding is convergent with literature data pointing out the potential role of SIRT1 in mediating the SGLT2i-related benefits in HF through downstream regulation of SGLT2 (Packer, 2020a; Packer, 2020e). SIRT1 (silent mating type information regulation 2 homolog) is a NAD + -dependent type III histone deacetylase, regulating several cellular processes by targeting transcription factors (Hwang et al., 2013). The downregulation of SIRT1 under high glucose levels might be associated with SGLT2 upregulation via GLUT2/importin-α1/HNF-1α pathway (Umino et al., 2018). Conversely, SIRT6 suppresses glucose metabolism by epigenetically silencing the HIF-1α (Liu et al., 2012). Our analysis using screening of the ENCODE transcription factor binding site profiles identified SIRT6 as a potential regulator of the SGLT2 activity highlighting its role as a potential co-regulator of the SGLT2 network.

The shortest path analysis between SGLT2 and key-terms-related genes highlighted the SGLT2 interactions with insulin, renin, albumin, adiponectin, and angiotensin I converting enzyme.

***SGLT2-INS*** (***INS target***) ***:*** The shortest path analysis demonstrated a strong interaction confidence level between SGLT2 and its target - INS among the top six from seven interactions associated with the key terms. In clinical trials, the inhibition of SGLT2 improved insulin sensitivity as explained by lowering insulin secretion from beta cells when the glucose concentration is decreased (Gharaibeh et al., 2019).

***REN-SGLT2*** (***REN as a mediator***) ***and ACE-SGLT2*** (***ACE target***) ***:*** We found the interaction between renin (REN) and SGLT2, where renin acts as a mediator. Additionally, there was detected interaction between angiotensin-converting enzyme (ACE), which was found as the target of SGLT2. Results of previous clinical trials suggested that glomerular hyperfiltration driven by the activation of SGLT2 and the renin-angiotensin-aldosterone system (RAAS) is the possible pathomechanism of diabetic nephropathy. SGLT2i reduces sodium reabsorption and increases sodium delivery to the macula densa, resulting in reduced renin release, followed by RAAS inhibition. They also regulate transport in all the tubule segments contributing to the reduction of oxygen demand, which improves the function of the epithelium (Nespoux and Vallon, 2018).

***ALB-SGLT2* (*ALB as mediator*)*:*** Shortest path analysis demonstrated an interaction between SGLT2 and ALB, acting as mediator. Albumins are responsible for the binding of the water, Ca (2+), Na (+), K (+), fatty acids, hormones, bilirubin, and drugs. It plays an important role in the regulation of the colloidal osmotic pressure of blood. Studies on mice showed SGLT2 overexpression in podocytes after bovine serum albumin injections and that SGLT2 inhibitor dapagliflozin limits podocyte damage in proteinuric nondiabetic nephropathy (Cassis et al., 2018). In humans, inhibition of SGLT2 leads to a reduction in albuminuria, which is essential for renal and cardiovascular protection (Bae et al., 2019).

***SGLT2-ADIPOQ* (*ADIPOQ as target*)*:*** Recent studies have shown that SGLT2i can also mediate body metabolism through regulation of adipokines levels, decreasing circulating leptin levels and increasing circulating adiponectin levels, which might contribute to the beneficial effects of SGLT2i on metabolic homeostasis (Wu et al., 2019).

### Identification of the Top SGLT2 Interactors

The shortest path analysis identified multiple genes known for their association with the SGLT2 network and SGLTi like SIRT1 and GLUT4. In this section, we decided to describe GLUT4 and less known top components of the SGLT2 network identified in this study, which appeared in the top 10 pathways analysis: ILK, ADCY7, GPT, COG2, and second level-interactions which level-interactions, which appeared in top 50 pathways analysis including MGAM. All top genes are shown in Table 1.

**GLUT4** encoded by SLC2A4 in the absence of insulin is efficiently retained intracellularly within storage compartments in muscle and fat cells. Mutations in this gene have been associated with noninsulin-dependent diabetes mellitus (NIDDM). GLUT4 is also a central player in hippocampal memory and brain insulin resistance (Reno et al., 2017; McNay and Pearson-Leary, 2020). Those results are convergent with our analysis showing that it is highly expressed in the brain. Other top interactors from the same family such as GLUT1 (Sayour et al., 2020) and GLUT2 (Ghezzi et al., 2018) have already been linked to SGLT2 (Scheepers et al., 2004).

**ILK** expression up-regulated by TGF-β in fibrosis has been proved to be a key mediator of TGF-β–induced fibrosis but this mechanism is still unknown (Li et al., 2009). A study on E-cadherin deficient mice model showed that ILK plays a role in mediating SGLT2-related kidney fibrosis in proximal tubular (Zheng et al., 2016). Taking into account that SGLT2 is directly regulated by TGF-β1 via Smad3 (Panchapakesan et al., 2013), TGF-β1 leads to decreased expression of SGLT1 and SGLT2 in renal proximal tubule cells (Lee and Han, 2010). Thus, these reports support the notion that ILK can play a role in SGLT2-related fibrosis.

**ADCY7** is mostly expressed in beta-cells, a calcium-sensitive protein, which converts ATP to cAMP. Loss of its function in RIN-m cell line led to a highly significant increase in mRNA levels of the insulin regulator genes Ins1 and Ins2 leading to the activation of the glucose stimulated-insulin secretion (GSIS) pathway (Alhaidan et al., 2021). ADCY7 also appeared in genome-wide identification of potential nephroprotective SGLT2i effects in human proximal tubular cells (Pirklbauer et al., 2019). Despite this, so far there is not much information regarding its interactions with SGLT2.

The number of studies associated with the **GPT** gene is highly limited. GPT gene encodes glutamate pyruvate transaminase, which might be involved in processes related to obesity. Abe et al. indicated that elevated GPT levels might reflect insulin resistance as well as act as a marker of metabolic syndrome in Japanese children (Abe et al., 2009).

**COG** encodes a subunit of the conserved oligomeric Golgi complex that is required for maintaining the normal structure and activity of the Golgi complex. Mutations of this gene are associated with abnormal glycosylation within the Golgi apparatus and its deficiency leads to hypoglycemia (Huang et al., 2021). A computational approach indicated that Golgi apparatus-associated genes are dysregulated in diabetes and identify putative markers of β-cell GA stress (Bone et al., 2020). So far, the information regarding COG interactions with SGLT2 and SGLT2i is very limited. Our results show that interaction can be important for further investigation.

**MGAM** main function is to digest terminal starch products left after the enzymatic action of α-amylase; hence, MGAM becomes a promising drug target for treating insulin resistance (Pałasz et al., 2019; Zhang et al., 2021). In our study, it appeared in six of the seven shortest paths analyses. Literature data does not provide information regarding its interactions with SGLT2, which makes it an interesting target for further studies.

Interpretation of the pathway enrichment analysis of the SGLT2 network components.

As expected, the pathway analysis revealed the strongest enrichment of the metabolism pathways. In various clinical trials, there were reports about the multidirectional effects of SGLT2i on human metabolism as shown in the introduction (Gager et al., 2021). SGLT2i affects transmembrane transport by decreasing Na+/H+ exchanger (NHE) activity in the cardiomyocyte’s cell membrane, which favorably affects their calcium balance and leads to the modulation of mitochondrial metabolism (Lopaschuk and Verma, 2020). The enrichment of energy metabolism was observed on both levels of the SGLT2 network in our study. We also observed enrichment of platelet signaling, activation, and aggregation involving ALB, PIK3R, and TIMP1 from shortest paths analysis. This result is convergent with a study showing that dapagliflozin-mediated atheroprotection in mice is driven by ameliorated thrombin–platelet-mediated inflammation and elevated HDL-cholesterol (Kohlmorgen et al., 2021). Additionally, analysis using the KEGG pathway database pointed out influence of the extended SGLT2 network on processes related to neuroactive ligand–receptor interaction and pathways in cancer. Genes involved in the neuroactive ligand–receptor interaction pathway, the major one for the activation of PI3K, are often upregulated in diabetes (Das and Rao, 2007). Through PI3K and AKT this pathway controls diverse biological processes such as programmed cell death, migration, proliferation, and inflammatory and metabolic processes. This result especially highlights how extended can be influence of SGLT2i on human organisms.

### Interpretation of the Disease Enrichment Analysis of SGLT2 Network Components

Disease enrichment analysis focused on the diseases associated with SGLT2 and revealed the strongest association with DM and hypertension (Figure 5). Those findings already have a connection proven in numerous publications and clinical trials involving SGLT2i (Zinman et al., 2015; Wiviott et al., 2019; Bhatt et al., 2021). Additionally, DM was also associated with SGLT1. As a novelty, we demonstrated an interaction between SGLT2 and malignant prostate and breast cancer. Zhou et al. showed that SGLT2 transporters are present in breast cancer cell lines. Additionally, the administration of dapagliflozin and canagliflozin resulted in a significant reduction of cell proliferation *in vitro*. In the mouse model of breast cancer, the administration of dapagliflozin decreased tumor growth, and the AMPK/mTOR pathway is considered a potential regulator of this (Zhou et al., 2020). Villani et al. indicated that SGLT2 inhibitor, namely canagliflozin inhibits mitochondrial complex-I resulting in reduced prostate cancer cell proliferation, which was presented in their *in vivo* and *in vitro* study (Villani et al., 2016). Our analysis revealed strong enrichment of SGLT2 and SGLT1 also for liver cancer. In the mouse model, the administration of canagliflozin resulted in a slower progression toward hepatocellular carcinoma (HCC). The authors indicate anti-inflammatory and anti-steatotic mechanisms of action of this SGLT2 inhibitor (Jojima et al., 2019). Additional analysis of biological processes associated with the extended SGLT2 network pointed out strong enrichment of regulation of inflammatory response. This provides a promising background for investigating the role of SGLTi in the context of inflammatory diseases, especially those associated with neutrophil dysfunctions. Such a beneficial influence of empagliflozin was observed before in treating inflammatory bowel disease in glycogen storage disease type Ib (Wortmann et al., 2020).

### Interpretation of the Overlap of Enrichment Analysis With SGLT2 Ligand Interactions

Overlap of enrichment analysis with SGLT2 ligands interactions pointed out four disease terms (diabetes mellitus, diabetes, hyperglycemia, diabetes mellitus non-insulin-dependent), which are associated with both SGLT2 and SGLT1 ligands. All of these terms refer to impaired glucose metabolism and diabetic conditions. SGLT2 and SGLT1 inhibitors have already proven therapeutic effects in those indications (Rieg and Vallon, 2018). Obtaining such results provides additional validation that the analysis was performed correctly.

Analysis of ligands targeting other strong components of the SGLT2 network besides SGLT2 or SGLT1 revealed that ertugliflozin, sergliflozin etabonate, and dapagliflozin have additional targets associated with hypertension. The benefits of SGLT2i in the context of blood pressure reduction were already observed. The potential mechanisms are Na + -H+ exchanger suppression and inhibition of overactivation of the sympathetic nervous system, which results in increased diuresis as well as reduction of arterial stiffness and vascular resistance (Kaplan et al., 2018; Wilcox, 2020). The analysis focused on dapagliflozin and identified all four targets as associated with diabetes mellitus ( namely SGLT1, SGLT6, SGLT2, and GLUT4). Our analysis revealed solid binding affinity and strong enzyme inhibition ability of dapagliflozin regarding SGLT2 > SGLT3 > SGLT1 > SGLT6 receptors (Figure 3). Dapagliflozin acts the most selectively regarding the SGLT2 transporter, which was confirmed by multiple studies (Demin et al., 2014). Interestingly, we observed that dapagliflozin also binds and decreases the activity of SGLT1 and SGLT3 receptors (specifically expressed in some segments of intestines), which may indicate its more extensive mechanisms of action (Subramaniam et al., 2019). Moreover, SGLT6 (known as SLC5A11 or SMIT2) is also one of dapagliflozin’s target receptors. Its inhibition is linked with a decrease in oxidative stress contributing to diabetic cardiomyopathy development (Van Steenbergen et al., 2017). Ertugliflozin and sergliflozin were regulating three of them ( namely SGLT1, SGLT2, and SGLT3). We hypothesize that this difference in the number of targets may affect the clinical response after using those SGLT2i.

For metformin, the highest number of its targets was associated with malignant neoplasm of the prostate (six genes) and breast carcinoma (seven genes). Several studies already emphasized metformin’s role in malignant prostate cancer (PC) (Margel et al., 2013; Spratt et al., 2013; Whitburn et al., 2017). The potential anticancer effect was observed in our study also on both levels of the whole SGLT2 interaction network, especially in the context of liver carcinoma.

### Limitations of the Study

The main limitation of the *in silico* study is its inclusivity. This approach often allows the identification of master regulators of the process targeting the highest number of genes overall but makes it difficult to identify highly specific biomarkers associated with rare phenotypes. To avoid this pitfall in this study we decided to split datasets into smaller parts (pathways, level of SGLT2 network regulation, tissues, and SGLT2i binding). It allowed us to more precisely filter out the top candidates based on multiple parameters. Another limitation was dealing with different nomenclature of the genes, especially coding SGLT proteins. To be able to merge and compare datasets across different databases we used the R function developed for this purpose based on NCBI nomenclature (Wicik et al., 2021). It enabled us to translate gene symbols into the most current ones before merging the datasets providing higher heterogeneity of how data were analyzed, annotated, and displayed. To avoid disparity between current knowledge we decided to use the String database and include all presented interactions, not only physical, assuming that some of them were not yet discovered but can be predicted based on other associations (co-expression, phylogenetics).

## Conclusion

The present article provides a comprehensive overview of the SGLT2 interaction network and its implications for specific effects of SGLT2i in order to explain its direct and indirect mechanism of action. SGLT2 has 65 first-level gene interactors and 5160 second-level interactors. Through bioinformatics analysis, we associated key terms and signaling pathways related to the SGLT2 in the context of tissue specificity and drug interactions. Using this approach, we identified remogliflozin as the most specific and dapagliflozin as the strongest inhibitor targeting SGLT2. The highest expression confidence of SGLT2 among tissues was observed in the kidney, liver, and adipose. The shortest pathway analysis identified SIRT1-SGLT2 among the top five interactions (autophagy, oxidative stress, aging, senescence, inflammation, and AMPK pathways). For the shortest path analysis, the strongest interaction confidences related to SGLT2 were ADIPOQ, INS, GLUT4, ACE, and GLUT1, but also ILK, ADCY7, DPP4, GPT, COG2, and MGAM. Pathway enrichment analysis showed that SGLT2 ligands have the strongest association with transport-related pathways and energy metabolism. We observed the highest enrichment in hypertension and DM-related diseases, and also in obesity and cancer-related diseases.

In conclusion, the exact mechanisms underlying SGLT2i effects have not yet been fully explained, even though the clinical outcomes in patients receiving SGLT2i showed the pleiotropic effect of the drugs. In this context, our analysis provides new and wide insight into this matter. SGLT2 showed significant interaction with multiple pathways, validating already known therapeutic effects and showing the potential as a therapeutic target in other diseases not yet treated with this class of drugs.

## Data Availability

The original contributions presented in the study are included in the article/Supplementary Material; further inquiries can be directed to the corresponding author.

## Supplementary Material

Supplementary File 1:

Supplementary Figure S1 Tissues sorted by the potential of being affected by SGLT2 and first-level SGLT2 interaction network.

Supplementary Figure S2 Tissues sorted by the potential of being affected by SGLT2i.

Supplementary Figure S3 Top 15 significantly enriched biological processes **(A)** and pathways **(B)** associated with Ist level SGLT2 network and first and second level SGLT2 interactors.

Supplementary Table S1 Association between diseases affected by SGLT2 network and targets of SGLT2 inhibitors.

Supplementary File 2: 1 excel file with following spreadsheets:
**key_terms**- listed ontological terms used in the analysis and databases used for their retieval. 
**GO_terms**- Gene ontology terms used for specific key terms. 
**genes_merged**- combined list of genes for all key terms and ontological terms used in the study. 
**SGLT2_network**- combined lists of genes used for creation 1st level and 2nd level SGLT2 network.

## Glossary

ABCB11: ATP Binding Cassette Subfamily B Member 11
ABCC2: ATP Binding Cassette Subfamily C Member 2
ABCC3: ATP Binding Cassette Subfamily C Member 3
ABCC4: ATP Binding Cassette Subfamily C Member 4
ACE: Angiotensin converting enzyme (ENSP00000290866)
ACE-001: Angiotensin converting enzyme 1 (ENSP00000387760)
ACSM5: Acyl-CoA Synthetase Medium-Chain Family Member 5
ADCY7: Adenylate Cyclase 7
ADIPOQ: Adiponectin, C1Q, and Collagen Domain Containing
AGXT2: Alanine-Glyoxylate Aminotransferase 2
ALB: Albumin
ALDH18A1: Aldehyde Dehydrogenase 18 Family Member A1
AMPK: 5′AMP-activated protein kinase
AQP2: Aquaporin 2
BP: blood pressure
BSA: Bovine serum albumin
CAB39: Calcium Binding Protein 39
CFAP57: Cilia and Flagella Associated Protein 57
CLCNKA: Chloride Voltage-Gated Channel Ka
CLCNKB: Chloride Voltage-Gated Channel Kb
CLTRN: Collectrin Amino Acid Transport Regulator
COG2: Component Of Oligomeric Golgi Complex 2
COX-2: Cyclooxygenase-2
CPB2: Carboxypeptidase B2
CREB3L3: CAMP Responsive Element Binding Protein 3 Like 3
CTRC: Chymotrypsin C
CV: Cardiovascular
DHFR: Dihydrofolate Reductase
DM: Diabetes mellitus
DPP4: Dipeptidyl Peptidase 4
DPP-4: Dipeptidyl peptidase 4
EGFR: Epidermal Growth Factor Receptor
FAM151A: Family With Sequence Similarity 151 Member A
FAP: Fibroblast Activation Protein Alpha
GCG: Glucagon
GIP: Gastric Inhibitory Polypeptide
GLP1R: Glucagon Like Peptide 1 Receptor
GLUT1: Glucose transporter 1
GLUT2: Glucose transporter 2
GLUT4: Glucose transporter 4
GPCRs: G protein-coupled receptors
GPT: Glutamic--Pyruvic Transaminase
GTEx: Genotype-Tissue Expression
HAUS3: HAUS Augmin like Complex Subunit 3
HbA1c: Higher glycated hemoglobin
HCC: Hepatocellular carcinoma
HDL: High-density lipoprotein
HF: heart failure
HG: High glucose
HIF: Hypoxia-inducible factor
HLA-A: Major Histocompatibility Complex, Class I, A
HLA-B: Major Histocompatibility Complex, Class I, B
HNF-1α: Hepatocyte nuclear factor-1α
IAPP: Islet Amyloid Polypeptide
IC50: half-maximal inhibitory concentration
IGT: Impaired glucose tolerance
IL-6: Interleukin-6
IL-8: Interleukin-8
ILK: Integrin Linked Kinase
ILVBL: IlvB Acetolactate Synthase Like
INS: Insulin
KCNJ1: Potassium Inwardly Rectifying Channel Subfamily J Member 1
Ki: Enzyme inhibition constant
KPTN: Kaptin Actin Binding Protein
KRAS: KRAS Proto-Oncogene, GTPase
LAD1: Ladinin 1
LAMTOR2: Late Endosomal/Lysosomal Adaptor, MAPK, and mTOR Activator 2
LIPC: Lipase C Hepatic Type
MAPK1: Mitogen-activated protein kinase 1
MAPK3: Mitogen-activated protein kinase 3
MFSD4E: Major Facilitator Superfamily Domain Containing 4E
MGAM: Maltase-Glucoamylase
MMP: Matrix Metallopeptidase
mTOR: Mechanistic Target Of Rapamycin Kinase
NADPH: Nicotinamide adenine dinucleotide phosphate
NARS: Asparaginyl-TRNA Synthetase
NF-kB: Nuclear factor kappa-light-chain-enhancer of activated B cells
NHE: Na+/H+ exchanger
NOX2: NADPH oxidase 2
PARP-1: Poly (ADP-ribose) polymerase 1
PC: Prostate cancer
PIK3R3: Phosphoinositide-3-Kinase Regulatory Subunit 3
PPI: Proton Pump Inhibitor
RAAS: Renin-angiotensin-aldosterone system
REG1A: Regenerating Family Member 1 Alpha
REN: Renin
RFS: Recurrence-free survival
RUSF1: RUS Family Member 1
SGLT1: Sodium-dependent glucose cotransporter type 1
SGLT2: Sodium-dependent glucose cotransporter type 2
SGLT2i: Sodium/glucose cotransporter-2 inhibitors
SI: Sucrase-Isomaltase
SIRT1: Sirtuin 1
SIRT6: Sirtuin 6
SLC12A1: (NKCC2) Solute Carrier Family 12 Member 1: (NKCC2) Solute Carrier Family 12 Member 1
SLC12A1: (NKCC2) Solute Carrier Family 12 Member 1: (NKCC2) Solute Carrier Family 12 Member 1
SLC12A3: (NCCT) Solute Carrier Family 12 Member 3
SLC13A3 (NADC3, SDCT2) Solute Carrier Family 13 Member 3
SLC13A4 (SUT1): Solute Carrier Family 13 Member 4
SLC15A2 (PEPT2): Solute Carrier Family 15 Member 2
SLC16A7 (MCT2): Solute Carrier Family 16 Member 7
SLC22A1 (OCT1): Solute Carrier Family 22 Member 1, ATP Binding Cassette Subfamily B Member 11
SLC22A2 (OCT2): Solute Carrier Family 22 Member 2
SLC22A8 (OAT3): Solute Carrier Family 22 Member 8
SLC25A21 (ODC): Solute Carrier Family 25 Member 21
SLC26A11: Solute Carrier Family 26 Member 11
SLC2A1 (GLUT1): Solute Carrier Family 2 Member 1
SLC2A11 (GLUT11): Solute Carrier Family 2 Member 11
SLC2A12 (GLUT12): Solute Carrier Family 2 Member 12
SLC2A14 (GLUT14): Solute Carrier Family 2 Member 14
SLC2A2 (GLUT2): Solute Carrier Family 2 Member 2
SLC2A3 (GLUT3): Solute Carrier Family 2 Member 3
SLC2A4 (GLUT4): Solute Carrier Family 2 Member 4
SLC2A5 (GLUT5): Solute Carrier Family 22 Member 8
SLC2A9 (GLUT9): Solute Carrier Family 2 Member 9
SLC3A1: Solute Carrier Family 3 Member 1
SLC47A1 (MATE1): Solute Carrier Family 47 Member 1
SLC5A1 (SGLT1): Solute Carrier Family 5 Member 1
SLC5A12 (SMCT2): Solute Carrier Family 5 Member 12
SLC5A2 (SGLT2): Solute Carrier Family 5 Member 2
SLC5A4 (SGLT3): Solute Carrier Family 5 Member 4
SLC6A18 (Xtrp2): Solute Carrier Family 6 Member 18
SLC6A19: Solute Carrier Family 6 Member 19
SLC6A2 (NET): Solute Carrier Family 6 Member 2
SLC7A13 (XAT2, AGT-1): Solute Carrier Family 7 Member 13
SLC9A3 (NHE3): Solute Carrier Family 9 Member A3
SMIT1: Sodium/myo-inositol transporter 1
SRC: Proto-Oncogene, Non-Receptor Tyrosine Kinase
T2DM: Type 2 diabetes mellitus
TIGAR: TP53 Induced Glycolysis Regulatory Phosphatase
TIMP1: TIMP Metallopeptidase Inhibitor 1
TPM: Transcripts Per Million
