# Supplementary material for: Characterization of the SGLT2 Interaction Network and Its Regulation by SGLT2 Inhibitors: A Bioinformatic Analysis
Source: Front Pharmacol. 2022 Aug 15;13:901340. doi: 10.3389/fphar.2022.901340 (PMC9421436; doi:10.3389/fphar.2022.901340)
Supplement: Supplementary file 1 [file DataSheet1.docx]

Supplementary Material

# Supplementary Figures and Tables

## Supplementary Figures


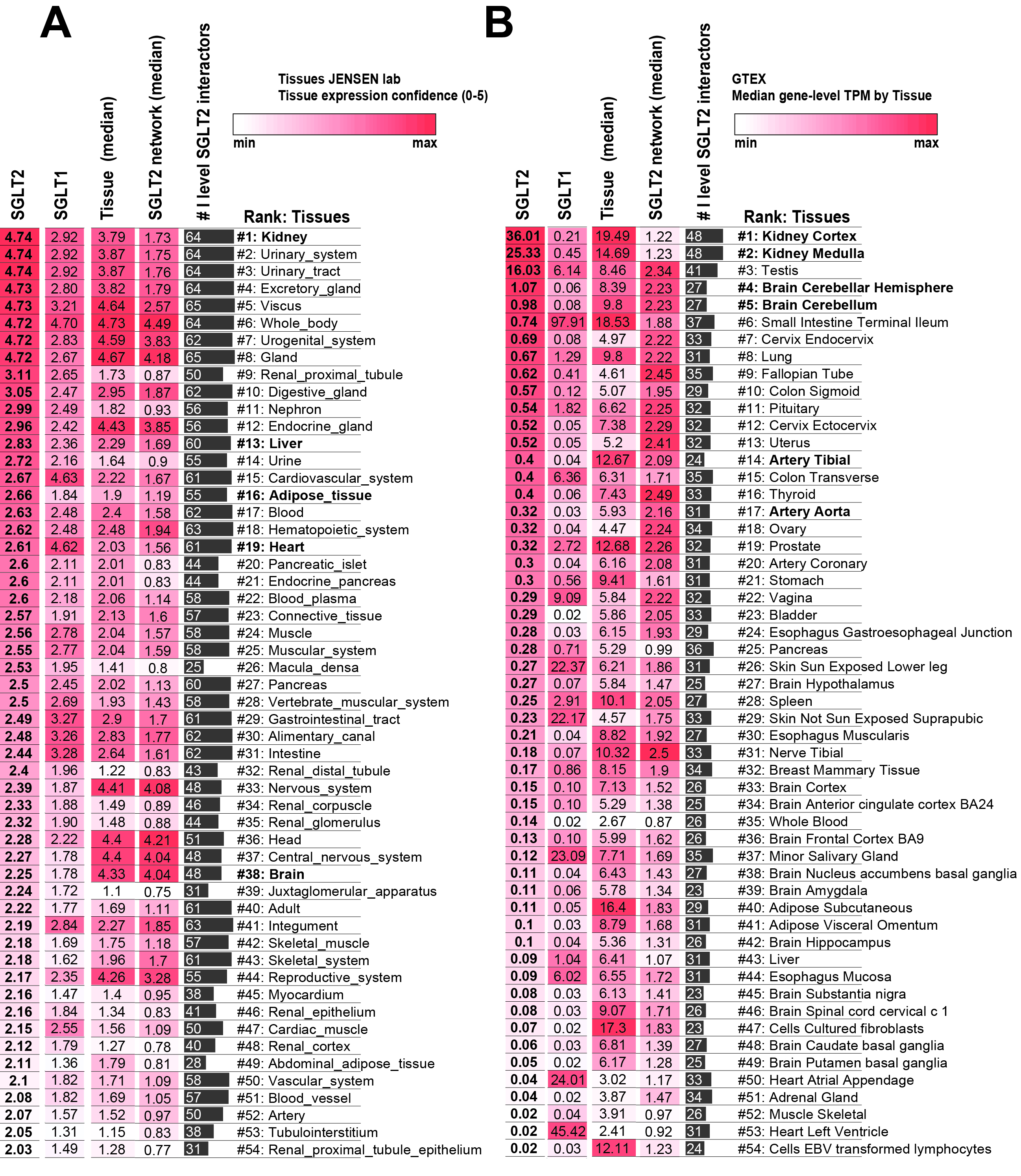


**Supplementary Figure 1.** Tissues sorted by the potential of being affected by SGLT2 and first-level SGLT2 interaction network. These lists of tissues were generated according to the concentration of SGLT2, obtained from (A) TISSUES 2.0 database expression confidence values, and (B) Genotype-Tissue Expression (GTEx) project Transcripts Per Million (TPM) values. On the figure are also present expression levels of SGLT1 which is a co-target of some SGLT2 inhibitors. Abbreviation: SGLT1, Sodium-dependent glucose cotransporter type 1; SGLT2, SGLT2, Sodium-dependent glucose cotransporter type 2


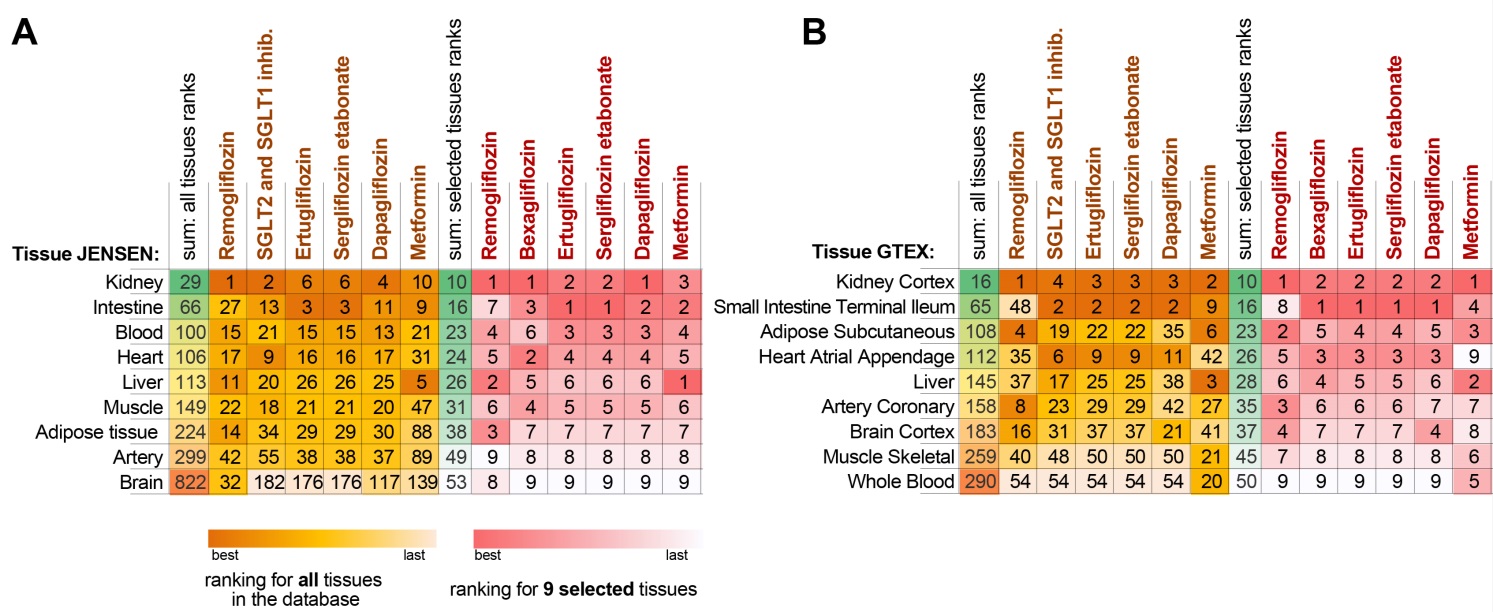


**Supplementary Figure 2.** Tissues sorted by the potential of being affected by SGLT2i. These lists of tissues were generated according to the concentration of drug targets, obtained from (A) TISSUES 2.0 database expression confidence values, and (B) Genotype-Tissue Expression (GTEx) project Transcripts Per Million (TPM) values. Tissues were ranked based on high targets number, high median expression and low standard deviation of the nodes.


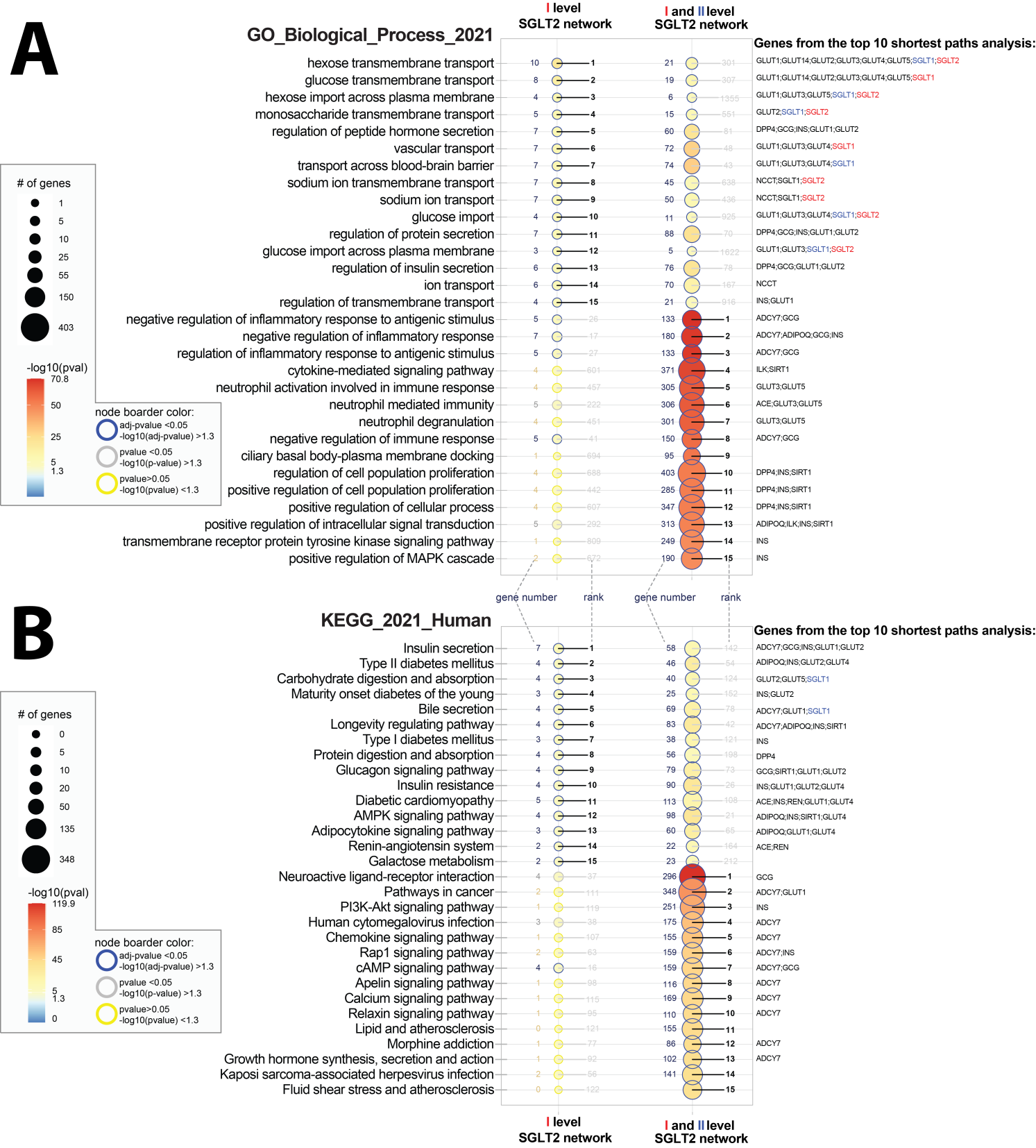


**Supplementary Figure 3.** Top 15 significantly enriched biological processes (A) and pathways (B) associated with first level SGLT2 network, and first and second level SGLT2 interactors. Analysis was performed using EnrichR API using the following databases GO_Biological_Process_2021 (biological processes) and KEGG_2019_Human (signaling pathways). The enriched terms were ordered by the level of significance for the first level SGLT2 network and then for the extended interaction network. SGLT2 (SLC5A2) is marked with red color, SGLT1 (SLC5A1, second-level interactor) with blue color. The adjusted p-values show categories which are more likely to have biological meanings. The color gradient of the dots is associated with corresponding adjusted p-values. Red color indicate low p-values (high enrichment), and blue indicate high p-values (low enrichment). The size of the dots is associated with the number of enriched genes.

## Supplementary Tables

**Supplementary Table 1.** Association between diseases affected by SGLT2 network and targets of SGLT2 inhibitors. Order of diseases follows figure 5.

| **Term** | **Remogliflozin** | **SGLT2 and SGLT1 inhibitors** | **Ertugliflozin** | **Sergliflozin etabonate** | **Dapagliflozin** | **Sophoraflavanone G** | **Kurarinone** | **Phlorizin** | **Phloretin** | **Metformin** |
| --- | --- | --- | --- | --- | --- | --- | --- | --- | --- | --- |
| Hypertensive disease | SGLT2 | *SGLT2* | SGLT2; SGLT3 | SGLT2; SGLT3 | SGLT2; SGLT3 | SGLT2 | ESR1; SGLT2 | SGLT2; SGLT3 | EGFR; GLUT1; SGLT2 | ABCC2; ABCC4; DPP4; SLC22A2 |
| Diabetes Mellitus | SGLT2 | SGLT1; SGLT2 | SGLT1; SGLT2; SGLT3 | SGLT1; SGLT2; SGLT3 | SGLT1; SGLT11; SGLT2; SGLT3 | BACE1; SGLT1; SGLT2 | BACE1; ESR1; SGLT1; SGLT2; SOAT1 | SLC29A1; SGLT1; SGLT2; SGLT3 | EGFR; GLUT1; SGLT1; SGLT2 | DPP4; SLC22A1; MATE1 |
| Diabetes | SGLT2 | SGLT1; SGLT2 | SGLT1; SGLT2; SGLT3 | SGLT1; SGLT2; SGLT3 | SGLT1; SGLT2; SGLT3 | BACE1; SGLT1; SGLT2 | BACE1; ESR1; SGLT1; SGLT2; SOAT1 | SLC29A1; SGLT1; SGLT2; SGLT3 | EGFR; GLUT1; SGLT1; SGLT2 | DPP4; SLC22A1; MATE1 |
| Hyperglycemia | SGLT2 | SGLT1; SGLT2 | SGLT1; SGLT2; SGLT3 | SGLT1; SGLT2; SGLT3 | SGLT1; SGLT2; SGLT3 | SGLT1; SGLT2 | ESR1; SGLT1; SGLT2 | SGLT1; SGLT2; SGLT3 | GLUT1; SGLT1; SGLT2 | DPP4 |
| Impaired glucose tolerance | SGLT2 | SGLT2 | SGLT2; SGLT3 | SGLT2; SGLT3 | SGLT2; SGLT3 | SGLT2 | ESR1; SGLT2 | SGLT2; SGLT3 | SGLT2 | DPP4 |
| Diabetes Mellitus, Non-Insulin-Dependent | SGLT2 | SGLT1; SGLT2 | SGLT1; SGLT2; SGLT3 | SGLT1; SGLT2; SGLT3 | SGLT1; SGLT2; SGLT3 | BACE1; SGLT1; SGLT2 | BACE1; ESR1; SGLT1; SGLT2; SOAT1 | SGLT1; SGLT2; SGLT3 | EGFR; GLUT1; SGLT1; SGLT2 | ABCC2; DPP4; SLC22A1; SLC22A2; MATE1 |
| Diabetic Nephropathy | SGLT2 | SGLT2 | SGLT2; SGLT3 | SGLT2; SGLT3 | SGLT2; SGLT3 | SGLT2 | ESR1; SGLT2 | SGLT2; SGLT3 | EGFR; GLUT1; SGLT2 | DPP4; SLC22A2 |
| Kidney Diseases |  |  |  |  |  |  | ESR1; SOAT1 |  | EGFR; GLUT1 | ABCC3; DPP4; SLC22A2; MATE1 |
| Diabetes Mellitus, Insulin-Dependent |  | SGLT1 | SGLT1 | SGLT1 | SGLT1 | SGLT1 | ESR1; SGLT1 | SGLT1 | EGFR; GLUT1; SGLT1 | ABCC2; SLC22A2 |
| Maturity onset diabetes mellitus in young |  |  |  |  |  |  |  |  | GLUT1 |  |
| Hypoglycemia | SGLT2 | SGLT2 | SGLT2; SGLT3 | SGLT2; SGLT3 | SGLT2; SGLT3 | SGLT2 | SGLT2 | SGLT2; SGLT3 | GLUT1; SGLT2 | DPP4 |
| Polyuria | SGLT2 | SGLT2 | SGLT2 | SGLT2 | SGLT2 | SGLT2 | SGLT2 | SGLT2 | SGLT2 |  |
| Hypokalemia |  |  |  |  |  |  |  |  |  |  |
| Renal Insufficiency |  |  |  |  |  |  |  |  |  | DPP4; SLC22A2 |
| Cardiovascular Diseases | SGLT2 | SGLT2 | SGLT2 | SGLT2 | SGLT2 | BACE1; SGLT2 | BACE1; ESR1; SGLT2; SOAT1 | SGLT2 | SGLT2 | DPP4 |
| Obesity |  | SGLT1 | SGLT1 | SGLT1 | SGLT1 | SGLT1 | ESR1; SGLT1; SOAT1 | SGLT1 | EGFR; GLUT1; SGLT1 | DPP4; SLC22A1; SLC22A2 |
| Liver carcinoma |  | SGLT1 | SGLT1 | SGLT1 | SGLT1 | CACNA1G; SGLT1 | ESR1; SGLT1; SOAT1 | SLC28A1; SGLT1 | EGFR; SLC16A3; GLUT1; SGLT1; TOP2A | ABCB11; ABCC2; ABCC3; ABCC4; SLC22A1 |
| Atherosclerosis |  |  |  |  |  |  | ESR1; SOAT1 |  | EGFR; SLC16A3 | DPP4; SLC22A1 |
| Malignant neoplasm of prostate |  |  |  |  |  |  | ESR1; SOAT1 |  | EGFR; SLC16A3; GLUT1; TOP2A | ABCC2; ABCC3; ABCC4; DPP4; SLC22A1; MATE1 |
| Breast Carcinoma |  |  |  |  |  | CACNA1G | ESR1; SOAT1 | SLC28A1 | EGFR; SLC16A3; GLUT1; TOP2A | ABCB11; ABCC2; ABCC3; ABCC4; DHFR; DPP4; SLC22A1 |
| Mammary Neoplasms |  |  |  |  |  |  | ESR1 | SLC28A1 | EGFR; GLUT1; TOP2A | ABCC2; ABCC3; DHFR; DPP4; SLC22A1 |
| Malignant neoplasm of breast |  |  |  |  |  | CACNA1G | ESR1; SOAT1 | SLC28A1 | EGFR; SLC16A3; GLUT1; TOP2A | ABCB11; ABCC2; ABCC3; ABCC4; DHFR; DPP4; SLC22A1 |
| Arteriosclerosis |  |  |  |  |  |  | ESR1; SOAT1 |  | EGFR; SLC16A3 | DPP4; SLC22A1 |
| Alzheimer's Disease |  |  |  |  |  | BACE1; CACNA1G | BACE1; ESR1; SOAT1 |  | EGFR; GLUT1 | ABCC2; ABCC4; DPP4 |
| Prostate carcinoma |  |  |  |  |  |  | ESR1; SOAT1 |  | EGFR; SLC16A3; GLUT1; TOP2A | ABCC2; ABCC3; ABCC4; DPP4; SLC22A1; MATE1 |
| Liver neoplasms |  |  |  |  |  |  | ESR1; SOAT1 |  | EGFR; GLUT1 | ABCB11; ABCC2; ABCC3; ABCC4; DHFR |
